# Central and peripheral apelin receptor distribution in the mouse: Species differences with rat

**DOI:** 10.1016/j.peptides.2011.12.005

**Published:** 2012-01

**Authors:** George R. Pope, Emma M. Roberts, Stephen J. Lolait, Anne-Marie O’Carroll

**Affiliations:** School of Clinical Sciences, Henry Wellcome Laboratories for Integrative Neuroscience and Endocrinology, University of Bristol, Bristol BS1 3NY, UK

**Keywords:** APJ, Apelin, *In situ* hybridization histochemistry, Autoradiography

## Abstract

The G protein-coupled apelin receptor (APJ) binds the endogenous peptide apelin and has been shown to have roles in many physiological systems. Thus far, distribution studies have predominantly been conducted in the rat and there is limited knowledge of the cellular distribution of APJ in mouse or human tissues. As recent functional studies have been conducted in APJ knock-out mice (APJ KO), in this study we undertook to characterize APJ mRNA and I^125^[Pyr^1^]apelin-13 binding site distribution in mouse tissues to enable correlation of distribution with function. We have utilized *in situ* hybridization histochemistry (ISHH) using APJ riboprobes, which revealed strong hybridization specifically in the paraventricular (PVN) and supraoptic (SON) nuclei of the hypothalamus and in the anterior pituitary, with marginally lower levels in the posterior pituitary. In the periphery, strong hybridization was observed in the lung, heart, adrenal cortex, renal medulla, ovary and uterus. Autoradiographic binding to APJ with I^125^[Pyr^1^]apelin-13 exhibited significant binding in the anterior pituitary, while lower levels were observed in the posterior pituitary and PVN and SON. In the periphery, strong receptor binding was observed in tissues exhibiting intense riboprobe hybridization, indicating a good correlation between receptor transcription and translation. While the distribution of APJ mRNA and functional protein in the mouse shows similarities to that of the rat, we report a species difference in central APJ distribution and in the pituitary gland.

## Highlights

► APJ is well characterized in the rat but is not well described in mouse tissues. ► We show the first detailed anatomical distribution of APJ mRNA and protein in the mouse. ► There is a species difference in central APJ distribution and in the pituitary gland. APJ distribution in peripheral tissues appears comparable between rat and mouse. ► The apelin system may have a more wide-ranging central role in the rat than the mouse.

## 1 Introduction

The recently described 36-amino acid peptide apelin [50] is associated with multiple biological actions in both the central nervous system (CNS) and in the periphery. In the CNS, apelin induces effects consistent with the regulation of body fluid homeostasis and stress responses [32,33,39], and also of cardiovascular [21] and central blood control [19]. In the periphery, the peptide is one of the most potent endogenous inotropic substances yet identified [49], and may modulate pulmonary function [22]. Unlike most other GPCR families apelin appears to mediate its effects via binding to only one receptor subtype, the previously orphaned apelin receptor (APJ). The APJ gene has a number of other aliases including APLNR, AGTRL1, APJR and FLJ90771, while the International Union of Pharmacology has recently recommended “apelin receptor” as the nomenclature for the receptor protein [37]. The cDNA sequences for human, mouse and rat APJ have been determined [10,34,36]. Rat APJ encodes a 377 amino acid polypeptide with a 96% and 89.7% overall amino acid identity with the mouse and human APJ respectively [34]. Other isoforms of the apelin peptide, including apelin-13 and the pyroglutamyl form of apelin-13 ([Pyr^1^]apelin-13), bind to and activate APJ and exhibit greater biological potency than the full-length peptide *in vivo* [50], yet in human cardiovascular tissues all three forms of apelin have comparable potency and efficacy [29].

The apelinergic system has a widespread expression and to date anatomical distribution studies have predominantly been conducted in the rat (see Table 1 for summary). Rat APJ mRNA distribution has been investigated using numerous techniques including *in situ* hybridization histochemistry (ISHH), Northern blots and reverse transcriptase-polymerase chain reaction (RT-PCR), with the strongest signals apparent in the lung and heart and lower levels evident in the brain hypothalamus and cerebroventricular region, pituitary gland, skeletal muscle, kidney, spinal cord, thyroid gland, adipose tissue, ovary and uterus [9,17,30,34]. Similarly, RT-PCR studies have shown widespread APJ mRNA expression in human tissues; high APJ expression was observed in human spleen, placenta, spinal cord and corpus callosum with lower levels present in the hypothalamus, hippocampus, lung, intestine, and stomach [30]. In contrast, quantitative real-time polymerase chain reaction (qPCR) studies in adult mouse tissues have shown APJ mRNA to be present in the pituitary, heart, lung, ovary, and uterus, with low expression levels in samples of whole brain and individual regions [30,41].

Limited distribution studies of APJ protein have been carried out to date. In the rat brain APJ protein expression was identified using immunohistochemistry (IHC) in the frontal and piriform cortices, the PVN, the pyramidal CA2 and CA3 cell layer of the hippocampus, dentate gyrus, spinal cord and cerebellum [9]. APJ immunoreactivity (APJ-ir) has also been shown in the SON and magnocellular vasopressin and oxytocin neurones of the pituitary [51] and in endothelial cells lining small intramyocardial, renal, pulmonary and adrenal vessels, small coronary arteries, large conduit vessels, and endocardial endothelial cells [21,24].

The regional localization and distribution of APJ led to further work clarifying the functions of this receptor. Thus high APJ expression in regions such as the heart and hypothalamic PVN and SON led to investigation of roles for APJ in the cardiovascular system and in the regulation of water balance and stress responses [8,21,27,31,49]. Recent studies have employed apelin- and/or APJ-knockout (KO) mice to further investigate the significance of the apelinergic system in cardiovascular function [19,25] and in fluid homeostasis [42,43]. APJ KO mice lack the hypotensive response to peripherally injected apelin that is seen in wild type littermates [19] and show a significant reduction in exercise capacity following exercise stress [8], suggesting roles for APJ in blood pressure regulation and cardiac function, respectively. Additionally APJ KO mice show abnormal water metabolism, manifested by a change in drinking behavior and in the ability to concentrate urine [42], and an altered response to the osmotic stress of salt loading [43] compared with wild type littermates, suggesting that APJ is an important regulator of mechanisms controlling fluid homeostasis. Furthermore apelin KO mice display an aging- and pressure overload-associated reduction in cardiac contractility [25] that supports the cardiovascular role of the apelinergic system. However (as illustrated in Table 1), while APJ is relatively well characterized in the rat it is not well described in an anatomical context in mouse tissues thus precluding correlations with function in these studies. Greater understanding of the distribution of APJ in the mouse will allow better insight and interpretation of results (describing function of the apelinergic system) from apelin- and APJ-KO mice. The aim of the present study was to provide an anatomical distribution of APJ mRNA and I^125^[Pyr^1^]apelin-13 binding sites in mouse brain and peripheral tissues to (1) determine whether mRNA encoding APJ corresponds to detectable functional protein (i.e. APJ processed and folded correctly to allow iodinated agonist binding); (2) determine whether there are species differences in APJ mRNA/protein expression between the mouse and rat; and (3) identify high expressing tissues in the mouse that may provide an anatomical basis for further experiments and understanding of the functions mediated by APJ and its cognate ligand. To date, no radiolabeled ligand has been used to comprehensively map the tissue distribution of APJ in any species. We have therefore used ISHH with riboprobes specific for the mouse APJ to detect APJ mRNA distribution and autoradiography with I^125^[Pyr^1^]apelin-13 to reveal apelin binding site localization.

## 2 Materials and methods

### 2.1 Animals

Male and female wildtype mice (8–14 weeks old, *n* = 6) from our APJ KO colony (a mix of the C57BL/6 and 129X1/SvJ strains) were used in this study [31,42]. Animals were housed under constant temperature (21 ± 2°C), light (lights on from 0700 to 1900 h) and humidity (45–50%) regimens with food and water *ad libitum*. Animal care and maintenance were performed in accordance with the Animal Scientific Procedures Act (1986) United Kingdom and the appropriate University of Bristol ethical review process.

### 2.2 *In situ* hybridization histochemistry (ISHH)

Sections (12 μm) of tissue were cut, thaw-mounted onto poly-lysine-coated slides (VWR, Lutterworth, UK) and stored at −80 °C until hybridization. All riboprobes were generated by PCR using 129sv genomic DNA as the template. For the mouse APJ ^35^S riboprobe primers (up-stream 5′-GCC CGA ATT CAC TTC ATT CAG CAC CAT GGA AGA T-3′; downstream 5′-GTC AGG ATC CCG GTA GGT ATA AGT GGC CCA CAG T-3′) corresponding to bp 256–549 of a mouse APJ cDNA (Genbank Accession number NM011784) were used to generate a 293 bp product. Primer restriction endonuclease sites allowed subcloning into pGEM4Z (Promega, Southampton, UK), and sense and antisense probes were generated using T7 and SP6 polymerases (antisense: linearized with EcoRI and generated with T7 polymerase; sense: linearized with HindIII and generated with SP6 polymerase) with ^35^S-UTP (Perkin Elmer, Cambridge, UK) and the MAXIscript *in vitro* transcription kit (Ambion, Huntingdon, UK). The integrity of each probe was verified by DNA sequencing.

Hybridization was carried out as described previously (http://intramural.nimh.nih.gov/lcmr/snge/Protocols/ISHH/ISHH.html). Slides were exposed to Amersham Hyperfilm MP film for 2 months at room temperature with appropriate ^14^C-labeled standards (Amersham, Little Chalfont, UK). No specific hybridization was detected with sense probes and no APJ mRNA signal above background was detected in tissues from APJ KO mice. Some slides were subsequently dipped in Ilford K5 nuclear emulsion and stored desiccated at 4 °C for 4–6 months before development using Kodak D19 at room temperature. Tissue sections were counterstained with toluidine blue.

### 2.3 Receptor autoradiography

Mouse cryostat sections (20 μm) were cut and thaw mounted onto subbed (gelatin, vanadium oxide) slides. APJ receptor autoradiography was performed with modifications of the procedure described by Katugampola et al. [21]. Brain sections were fixed in 0.1% PFA in PBS for 5 min and rinsed in 10 mM Hepes pH 7.5. All sections were pre-incubated for 20 min in 20 mM Hepes pH 7.5 containing 1 mM EDTA, 0.3% BSA and Sigma Protease Inhibitor Complex (Sigma, Dorset, UK). Slides were then incubated in 20 mM Hepes, pH 7.5, 100 mM NaCl, 5 mM MgCl_2_, 10 mM KCl, 1 mM EDTA, Sigma protease inhibitor complex and 0.3% BSA containing radiolabeled (Glp^65^, Nle^75^, Tyr^77^) (^125^I)-Apelin-13 [0.5 nM] (Perkin Elmer, Cambridgeshire, UK) in the absence or presence of unlabeled (Pyr^1^)-apelin-13 [1 μM] (Bachem, Germany) as a displacer. Binding specificity was assessed by comparison of the distribution of [^125^I]-(Pyr^1^)apelin-13 binding sites in wildtype tissue to that in APJ KO tissue. Incubation lasted 1 h at RT in a humid chamber and was followed by 2 × 10 min washes in ice-cold 20 mM Hepes pH 7.5, 0.3% BSA with stirring and 2 × 15 min washes in ice-cold 10 mM Hepes pH 7.5. Slides were then rinsed in ice-cold dH_2_O and air-dried at 4 °C before being exposed to X-ray film (Amersham Hyperfilm MP) for 2 weeks. Following this some slides were re-exposed to emulsion-coated film (Amersham Hyperfilm ^3^H) for 1 month to obtain better macroscopic resolution. Films were developed as described for ISHH, except emulsion-coated films, which were developed manually as per manufacturer's instructions.

## 3 Results

### 3.1 APJ mRNA distribution in the mouse

**Fig. 1:**
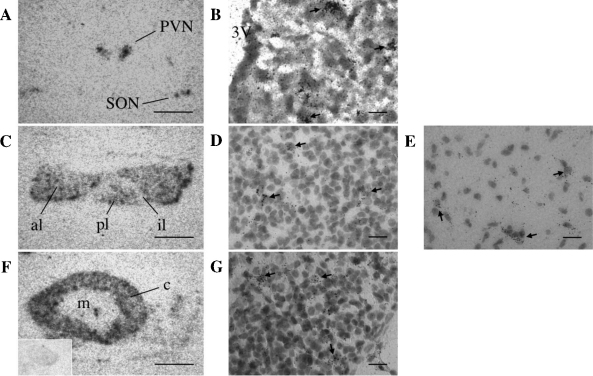
Localization of APJ mRNA in the HPA axis of the adult mouse by ISHH. Low magnification images of autoradiographic films showing slide mounted tissue sections hybridized with an antisense ^35^S-labeled APJ riboprobe. (A) Labeling of the hypothalamic paraventricular (PVN) and supraoptic (SON) nuclei; (B) shows bright-field high power micrographs of APJ mRNA distribution within the mouse PVN. (C) Signal is present in populations of cells in the anterior lobe (al) and posterior lobe (pl) of the pituitary, however very few cells expressing APJ mRNA were detected in the intermediate lobe (il); (D) and (E) are bright-field high power micrographs of APJ mRNA distribution in the anterior and posterior lobes of the pituitary, respectively. (F) In the adrenal gland strong signal is seen in all regions of the cortex (c) with no signal detected in the medulla (m). Image from corresponding sense ^35^S-labeled APJ riboprobe in mouse adrenal gland is shown (inset); (G) shows bright-field high power micrographs of APJ mRNA distribution in the adrenal gland. Scale bars in A, C and F equal 1 mm, scale bars in B, D, E and G equal 100 μm.

**Fig. 2:**
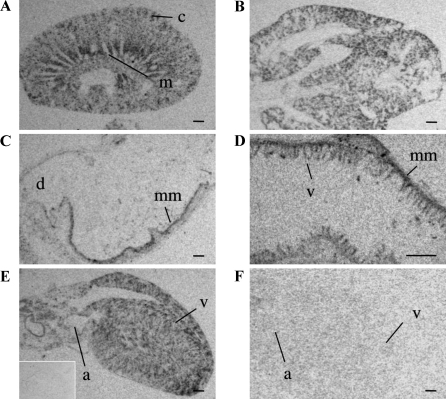
Localization of APJ mRNA in the peripheral tissues of the adult mouse by ISHH. Low magnification images of autoradiographic films of slide-mounted tissue sections hybridized with an antisense ^35^S-labeled APJ riboprobe. (A) The kidney exhibits labeling in the outer cortex (c) and the medulla (m). (B) In the lung there is strong labeling throughout the parenchyma. (C) The pyloric region of the stomach is shown with specific labeling in the mucosa muscularis (mm) and the duodenum indicated (d). (D) At higher magnification, specific labeling in the villi (v) and mucosa (mm) of the ileum is observed. (E) Labeling in the heart atrium (a) and ventricle (v) with corresponding sense ^35^S-labeled APJ riboprobe shown (inset). (F) An absence of signal was observed in the APJ KO heart atrium (a) and ventricle (v). All scale bars equal 1 mm.

**Fig. 3:**
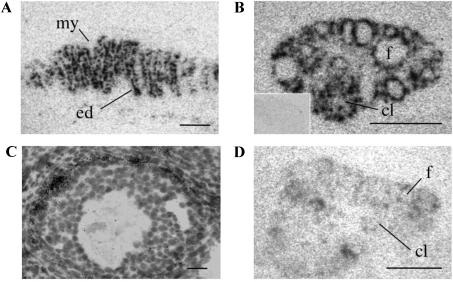
Localization of APJ mRNA in the periphery of the adult mouse by ISHH. Low magnification images of autoradiographic films of slide mounted tissue sections hybridized with an antisense ^35^S-labeled APJ riboprobe. (A) Labeling of cells of the endometrial layer of the uterus (ed) with no labeling of the myometrium (my). (B) Labeling present in an ovary, with follicle (f) and corpus luteum (cl) indicated, with sense ^35^S-labeled APJ riboprobe shown (inset); (C) is a brightfield high power micrograph of an emulsion dipped section showing APJ mRNA distribution within a follicle. (D) APJ KO ovary with follicle (f) and corpus luteum (cl) indicated. Scale bars in A, B and D equal 1 mm, scale bar in C equals 100 μm.

ISHH with antisense APJ riboprobes was used to map the distribution of APJ mRNA in the male and female mouse brain and peripheral tissues. Sections from all tissues were also hybridized with sense APJ riboprobes as controls and showed only background level of labeling. A number of tissues, including the pituitary, lung, heart, ovary and uterus, showed high levels of hybridization, with representative photographs shown in Figs. 1–3. Within the brain APJ mRNA had a very restricted distribution where the PVN and SON hypothalamic regions showed high levels of gene expression (Fig. 1A and B). No labeling of other structures throughout the brain was observed. Within the other components of the hypothalamic–pituitary–adrenal (HPA) axis, the pituitary gland showed high to moderate expression of APJ mRNA in the anterior and posterior lobes respectively, where expression was found in scattered cells with some sparse signal in the intermediate lobe (Fig. 1C–E). The cortex of the adrenal gland also showed prominent hybridization of the three cortical zones with no expression seen in the medulla (Fig. 1F and G).

In the kidney a high level of APJ expression was seen in the medulla, specifically the inner stripe of the outer medulla, consistent with hybridization to the medullary rays, with patch-like labeling observed in the outer cortex that may correspond to tubular structures (e.g. distal/proximal tubule) (Fig. 2A). No labeling was seen in the glomeruli. In the lung, APJ mRNA was restricted to the parenchyma (Fig. 2B) and there was no evidence of any association with the lining of blood vessels or in the bronchi or bronchioles. In the pyloric region of the stomach the mucosal layer of the stomach lining showed a strong hybridization signal for APJ (Fig. 2C) with transcript also seen within the villi of the ileum (Fig. 2D). Hybridization within the heart was widespread with APJ expression present in cardiomyocytes throughout the myocardium (Fig. 2E). No signal was observed in heart sections hybridized with sense probe (inset), similarly no APJ mRNA signal was detected in heart tissue from APJ KO mice (Fig. 2F).

Moderate hybridization levels were present in the uterine endometrial lining, however no signal was detected in the myometrium (Fig. 3A). In the ovary (Fig. 3B), the theca cells surrounding the antral follicles showed intense labeling (Fig. 3B and C) as did the cells of the corpus luteum (Fig. 3B), while no signal was present in ovary sections hybridized with sense probe (Fig. 3B, inset) and only background levels of radiolabeling were detected in the ovary of APJ KO mice (Fig. 3D).

APJ mRNA, as indicated by the presence of hybridization signal, was not detected in a number of other tissues including liver, spleen, gall bladder, thymus, trachea, pancreas and testis (images not shown). The pattern of APJ mRNA expression was similar between male and female mice. The data is summarized in Table 1.

### 3.2 Receptor autoradiography

**Fig. 4:**
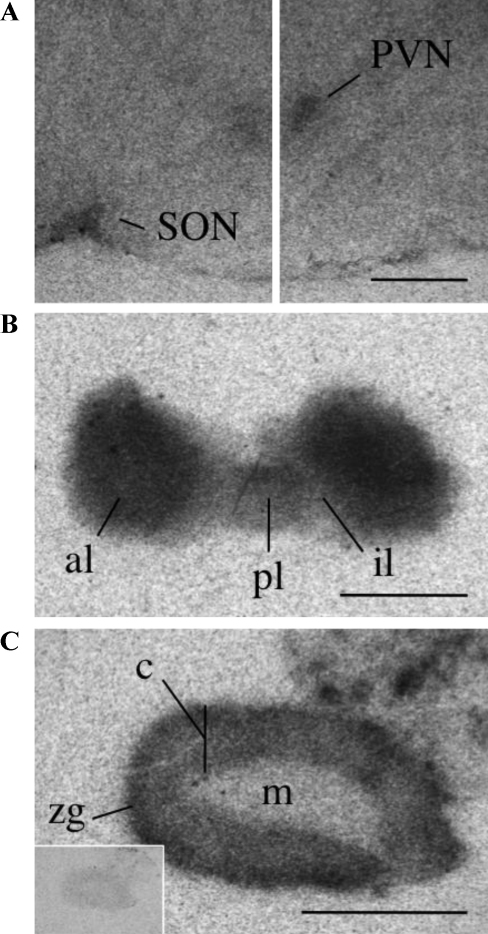
Representative autoradiographical localization of ^125^I-[Pyr^1^]apelin-13 binding in the HPA axis of the adult mouse. (A) Individual sections displaying specific binding of ^125^I-[Pyr^1^]apelin-13 (0.5 nM) in the hypothalamic PVN and SON. (B) The anterior pituitary (ap) is heavily labeled, with moderate levels of binding localized to the posterior lobe (pl) and non-specific levels seen in the intermediate lobe (il). (C) The cortex (c) of the adrenal gland displays strong binding with the highest density seen in the outer zona glomerulosa (zg) with an absence of specific binding sites in the presence of [1 μM] unlabeled (Pyr^1^)apelin-13 (inset). All scale bars equal 1 mm.

[^125^I]-(Pyr^1^)apelin-13 was used to localize APJ binding sites in the mouse brain and peripheral tissues. Binding specificity was assessed by binding of radiolabeled apelin-13 in the presence of 1 μM unlabeled (Pyr^1^)apelin-13 and by comparison of APJ distribution in wildtype mouse tissue to that in APJ KO tissues, where no specific binding was observed in any tissue. Of note, while APJ binding corresponds to correctly processed and folded receptors it does not unquestionably infer that the receptors present are capable of signaling. In agreement with our localization results obtained using ISHH in wildtype mice, strong binding of [^125^I]-(Pyr^1^)apelin-13 was observed in the pituitary, lung, heart, adrenal gland, ovary and uterus, indicating high expression of APJ, with lower densities in the brain and bladder, while no binding was in seen in the liver, pancreas and testis. In the hypothalamus binding was localized to the PVN and SON (Fig. 4A). No binding of other structures throughout the brain was observed. High densities of APJ were present in the anterior lobe of the pituitary with moderate levels of binding sites seen in the posterior lobe. Little to no binding above background levels was seen in the intermediate lobe (Fig. 4B). [^125^I]-(Pyr^1^)apelin-13 binding was also seen in the adrenal cortex with the highest receptor densities seen in the zona glomerulosa and no APJ binding sites were found in the medulla (Fig. 4C). No binding was detected in the adrenal gland in the presence of unlabeled ligand (inset Fig. 4C).

**Fig. 5:**
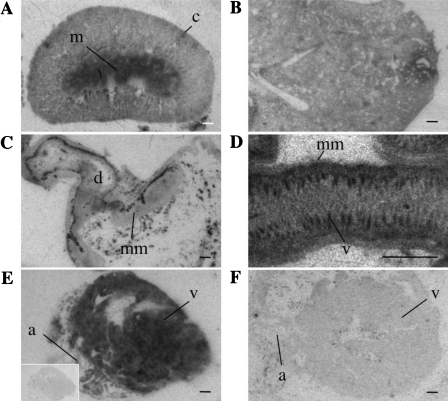
Representative autoradiographical localization of ^125^I-[Pyr^1^]apelin-13 binding in peripheral tissues of the adult mouse. (A) In the kidney low concentrations of ^125^I-[Pyr^1^]apelin-13 binding sites are present in the cortex (oc) and high densities of APJ in the medulla (m). (B) Binding to lung parenchyma (C) The pyloric region of the stomach exhibits binding in the mucosa muscularis (mm) surrounding the duodenum (d). (D) The ileum shows strong binding in the villi (v) and to a lesser extent in the mucosa (mm). (E) The heart, atria (a) and ventricles (v) show strong binding to APJ, with lack of binding in the presence of [1 μM] unlabeled (Pyr^1^)apelin-13 (inset). (F) Absence of specific receptor binding sites in the APJ KO heart atrium (a) and ventricle (v). All scale bars equal 1 mm.

In the kidney the most dense localization of [^125^I]-(Pyr^1^)apelin-13 binding sites was found in the outer medulla with patches of binding found in the cortex (Fig. 5A). The lung showed uniform binding to the parenchyma with no binding sites detected in connective tissue or blood vessels (Fig. 5B). High densities of APJ binding sites were localized to the mucosal layer of the pyloric region of the stomach (Fig. 5C) as well as in the mucosa and villi of the ileum (Fig. 5D). The density of APJ binding sites in the heart was uniform throughout the myocardium (Fig. 5E). No specific binding was detected in the presence of unlabeled ligand (Fig. 5E, inset) not in the heart of APJ KO mice (Fig. 5F).

**Fig. 6:**
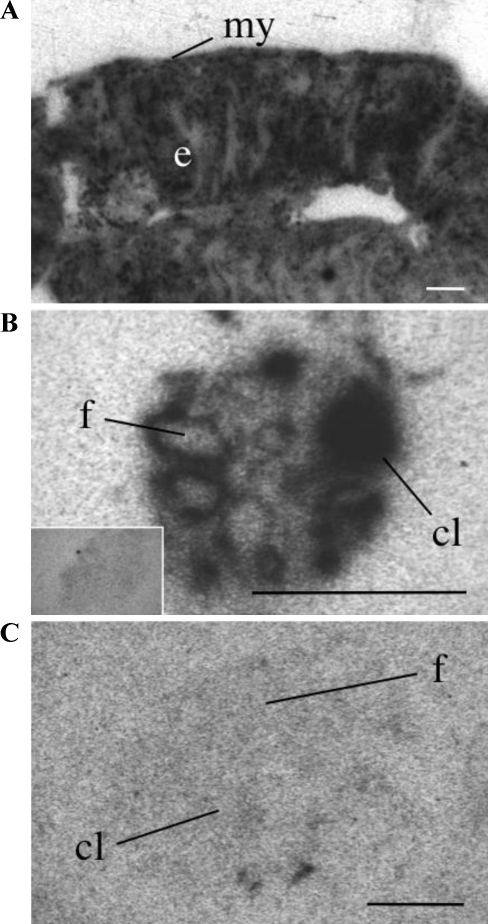
Representative autoradiographical localization of ^125^I-[Pyr^1^]apelin-13 binding in peripheral tissues of the adult mouse. (A) Specific binding of ^125^I-[Pyr^1^]apelin-13 (0.5 nM) in the uterine endometrium (ed) and (B) the follicle (f) and corpus luteum (cl) of the ovary, with an absence of binding in the ovary in the presence of [1 μM] unlabeled (Pyr^1^)-apelin-13 (B inset). (C) Absence of binding in the APJ KO ovary follicle (f) or corpus luteum (cl). Myometrium (my). All scale bars equal 1 mm.

In the uterus very high levels of binding were present in the endometrium but totally absent from the myometrium (Fig. 6A). The ovary displayed strong binding in the theca cells of follicles and in corpus lutea (Fig. 6B) while no binding occurred in the presence of unlabeled (Pyr^1^)apelin-13 (Fig. 6B, inset), Specific labeling of (Pyr^1^)apelin-13 binding sites was absent in the APJ KO ovary (Fig. 6C).

## 4 Discussion

Previous studies mapping APJ distribution have focused primarily on APJ mRNA expression in rat brain and peripheral tissues and few studies have investigated the distribution of APJ protein in any species. The present study provides the first detailed characterization of APJ mRNA and I^125^[Pyr^1^]apelin-13 binding site distribution in the mouse. We have found that APJ mRNA and I^125^[Pyr^1^]apelin-13 binding site localization appear to be unaffected by gender and that there is a clear correlation between the expression of APJ mRNA and I^125^[Pyr^1^]apelin-13 binding. A summary of our findings is shown in Table 1.

### 4.1 CNS

We report a restricted localization of both APJ mRNA and I^125^[Pyr1]apelin-13 binding sites in the mouse CNS, with discernable levels found only in the hypothalamic PVN and SON. While we cannot discount that the level of APJ in additional regions of the mouse CNS is too low to allow detection by the techniques used in our study, comparable studies in rats have revealed high levels of APJ mRNA in the cerebroventricular system, hypothalamus, the pineal gland, olfactory bulb and hippocampus [9,17,34], suggesting a species difference in central APJ distribution. Furthermore, RT-PCR studies on human samples confirm the widespread APJ mRNA expression in the CNS, with highest levels in the spinal cord and corpus callosum and with lower expression detected in the hypothalamus and hippocampus [30]. The central apelinergic system in rats appears to be involved in cardiovascular regulation [20] and activation of the arcuate POMC network [40]. It also appears to protect the hippocampus from excitotoxicity, including that induced by human immunodeficiency virus type I [35]. In mice, immediate early gene expression in the subfornical organ, median preoptic nucleus and PVN in response to perturbations in water homeostasis is altered in APJ-KO mice [42,43]. In addition, central apelin administration in mice increases CRF- and VP-induced ACTH secretion [31], regulates energy homeostasis [11,52], inhibits gastric emptying and gastrointestinal transit [28] and has antinociceptive effects [54]. Many of these central effects are thought to be mediated at the level of the hypothalamus. The functional significance of the apparent species differences in the central expression of APJ mRNA is not known. Profound species differences in central GPCR expression is not uncommon – a striking example is the pattern of oxytocin and VP receptor expression in rodents [3] which may provide the anatomical substrates for species differences in the expression of social behavior. There appear to be differences in the meningeal and hippocampal expression of APJ between mice and rats (e.g., see Fig. 2 in Hazell et al. [16]). As APJ can potentially act as a co-receptor for viruses in non-immune cells [38,46], one intriguing possibility is that species or strain differences in meningeal cell and hippocampal APJ expression levels may influence the susceptibility to certain microbes and contribute to neuroprotection, respectively.

### 4.2 Pituitary

In the pituitary gland of the mouse high to moderate APJ mRNA expression was observed in cells of the anterior and posterior lobes respectively with only sparse labeling in the intermediate lobe. This differs from the rat with reports of a moderately strong distribution of APJ mRNA in the anterior lobe but not in the posterior or intermediate lobes [34]; or as shown by De Mota and co-workers [9], APJ mRNA expression in the anterior and intermediate lobes but not in the posterior lobe of the rat pituitary. In contrast APJ-ir has been found in the nerve terminals of the rat posterior pituitary gland [51]. Our study in mice suggests that APJ mRNA is present in an unidentified posterior pituitary cell type that may be resident pituicytes or glial cells where other GPCRs including the V1a receptor are known to be expressed and speculated to indirectly influence neurohypophysial hormone release [15]. The extent of APJ binding sites, i.e. widespread rather than restricted to scattered cells, could suggest that APJ is expressed in both cells and nerve terminals in the mouse posterior pituitary. The SON and PVN are well-known integrative sites that regulate coordinated responses to osmotic stressors. Vasopressin, along with oxytocin, is synthesized primarily within these neurones, which project their axons from hypothalamic cell bodies to terminals on the capillaries of the posterior pituitary neural lobe to release the peptides into the systemic circulation. Hormone release studies from isolated rat SON and neural lobes *in vitro* show significantly decreased basal vasopressin release from SON but not from neural lobe preparations after apelin administration, indicating a possible role for apelin in dendritic rather than axonal vasopressin release [51]. The species difference in APJ distribution seems likely to reflect a more extensive role for apelin in mouse pituitary function to regulate neurohypophysial hormone release.

### 4.3 Adrenal gland

In the mouse adrenal gland APJ mRNA and I^125^[Pyr^1^]apelin-13 binding sites were expressed throughout the cortex, with little to no presence in the medulla. This is the first reported detailed distribution of APJ expression within the rodent adrenal gland, with no described function to date. The localization however, points to a possible role of APJ and its cognate ligand in corticosteroid release. In contrast to the mouse distribution, in human adrenals APJ-ir is confined to endothelial cells of the surrounding arteries, small resistance arteries within the capsular plexus and the central vein while APJ-ir was not detectable in secretory cells of either the adrenal cortex or medulla [23].

### 4.4 Kidney

APJ mRNA and I^125^[Pyr^1^]apelin-13 binding sites were present throughout mouse renal cortex and medulla, however APJ expression was not integral to the glomeruli as previously reported in the rat [34], a localization that was suggestive of a role for this receptor in the regulation of blood flow or glomerular filtration. Expression in the mouse was associated with the renal corpuscle, similarly signal was observed in sporadic cells along proximal and distal tubules although a specific association with blood vessels or collecting ducts, as has been seen previously in the rat [18], was not observed. The low resolution of APJ mRNA on autoradiographic films of the kidney does not allow us to clarify morphologically the exact cell types in the kidney within which mouse APJ expression is localized. APJ mRNA has also been identified in mouse kidney using RT-PCR [30]. The role of apelin in the kidney is unclear however strong expression of APJ mRNA and high levels of I^125^[Pyr^1^]apelin-13 binding suggests an involvement of peripheral aspects of the apelinergic system in the regulation of fluid homeostasis as has been suggested by studies in the APJ KO mouse [42,43], while APJ expression in the highly vascularized inner stripe of the outer medulla suggests a possible renal medullary microcirculatory role for the mouse receptor. In the human APJ mRNA expression is found in the kidney [30], while in sections from human kidney APJ-ir is present in endothelial cells and in vascular smooth muscle cells of small intrarenal vessels [23].

### 4.5 Lung

In the lung APJ mRNA was detected in the parenchyma as previously described in the rat [10], while unlike recent reports of APJ distribution in the rat, there was no evidence of expression in the lining of pulmonary blood vessels [1]. Additionally no expression was seen in endothelial and vascular smooth muscle cells from small pulmonary vessels as reported for rat and human lung [24]. The strong expression of APJ in the lung suggests that it plays a significant, though yet undescribed, role in pulmonary function

### 4.6 Stomach and intestine

APJ mRNA distribution in the mouse stomach was predominantly within the glandular region and ‘body’ of stomach, not the fore stomach, in agreement with RT-PCR results reported in the rat by Hosoya and co-workers [17]. In the intestine, APJ mRNA and I^125^[Pyr^1^]apelin-13 binding sites are localized to the mucosa and more evidently to the villi. Apelin has previously been shown to stimulate the secretion of cholecystokinin (CCK), responsible for stimulating the digestion of fat and protein, from a murine small-intestinal cell line (STC-1) [53], and to be present in luminal perfusate of the rat intestine. While CCK cells are present in the duodenum and jejunum of the intestine [26], APJ has not been localized to CCK cells. However, these recent studies suggest that APJ may be found on CCK cells, facilitating apelin stimulation of CCK secretion [53].

### 4.7 Heart

In our study *in situ* hybridization signal and receptor binding within the mouse heart were widespread, with APJ expression found predominantly throughout the myocardium with minimal signal associated with vessels. A high level of APJ mRNA expression was detected by quantitative RT-PCR in the rat heart [17] and these findings were confirmed by Northern blot analysis and ISHH [34]. APJ has been detected in rat and human myocardium as well as in the medial layer of human coronary artery, aorta and saphenous vein using radioligand binding [51], and APJ-ir is present in endothelial cells, vascular smooth muscle cells and cardiomyocytes [30]. Recent studies indicate a role for the apelin–APJ signaling pathway in basic cardiac function and during the development of hypertension and there is growing evidence that apelin may be involved in the transition from compensated hypertrophy to clinically significant heart failure [12]. Apelin may therefore act as a cardiovascular regulator in the human and rat, and it has been shown to have a sustained positive inotropic effect on intact rat hearts [2,4,49], with a more transient effect observed in *ex vivo* myocytes [13]. Thus, expression of APJ transcripts and protein in the mouse cardiomyocytes supports the proposed cardiovascular effects of apelin.

### 4.8 Uterus and ovary

High levels of APJ mRNA and I^125^[Pyr^1^]apelin-13 binding sites were detected throughout the mouse uterine endometrium. There was no evidence of APJ expression within endometrial glands or in the myometrium, consistent with APJ mRNA distribution in the rat uterus [34]. In agreement with these findings a comparatively high level of APJ mRNA expression was detected by quantitative RT-PCR in the mouse uterus when compared with rat and human tissue [17].

In the mouse ovary APJ mRNA and I^125^[Pyr^1^]apelin-13 binding were predominantly associated with theca cells surrounding antral follicles. APJ was not localized around primary follicles, nor associated with the major vasculature within the interstitium. Numerous sporadic cells with a very dense expression of APJ mRNA were located throughout the interstitium. The corpus luteum had a high level of expression of APJ – the pattern of expression is consistent with the distribution of the theca lutein cells formed from the theca-interna following rupture of the follicle. In the rat ovary intense labeling was observed in cells located toward the periphery of the corpora lutea mass and in some theca and stromal cells surrounding large antral follicles, while granulosa and theca cells of small antral follicles, theca lutein and interstitial cells did not express APJ mRNA [34]. APJ and apelin mRNAs have a distinct but overlapping distribution in the rat ovary. All corpora lutea express high levels of APJ mRNA but not all APJ mRNA expressing corpora lutea contain apelin mRNA (O’Carroll, unpublished observation) – whether this is related to the stage of the corpora lutea (e.g. new or regressing) has not been established. The distribution of mRNA encoding APJ and apelin within the ovary is suggestive of a role for apelin as a novel modulator of ovarian function. The expression of both apelin and APJ mRNAs in some corpora lutea and theca cells suggests that the intraovarian apelin system may have an autocrine role. In addition, a paracrine action of apelin is supported by the demonstration of both apelin and APJ gene expression within the same subset of luteal cells [47]. In particular, the prominent localization of the apelin/APJ system in corpora lutea suggests that it may participate in luteolysis, vascularization and/or regression/apoptosis within this compartment. Data from bovine ovary suggest that apelin/APJ system is involved in the mechanism regulating angiogenesis during follicle maturation as well as during corpora lutea formation [44] and there is also evidence of APJ and apelin in theca and granulosa cells participating in follicular atresia [45].

Apelin has been found to have as extensive a distribution as APJ and in all of the tissues examined in this study, where we have found the greatest expression of APJ in the mouse, apelin has also been reported to be present. In regions such as the PVN/SON and the anterior and posterior lobes of the pituitary apelin distribution is very similar to that of APJ [6,22,27,30]. Studies on apelin distribution in peripheral tissues are limited, with whole tissue distribution studies exhibiting high levels of apelin in mouse lung, heart, kidney and ovary [30]. Such matched distributions could indicate the presence of autocrine or paracrine signaling systems in these tissues.

No expression of APJ transcript or I^125^[Pyr^1^]apelin-13 binding was observed in a number of mouse tissues including liver and pancreas. Using RT-PCR however, APJ mRNA has been identified in mouse and human liver and pancreas [30,41,48]. Apelin has been identified as a novel adipokine, which is upregulated by obesity and hyperinsulinemia in both humans and mice [5,7]. Expression of APJ in mouse islets has been reported where apelin-36 inhibited glucose-stimulated insulin secretion both *in vivo* and *in vitro* [48] suggesting a link between this adipokine and glucose homeostasis. Thus, apelin may be involved in the regulation of islet function, although its precise role remains to be established.

Additionally the finding that APJ is not expressed in mouse testis is intriguing as moderate levels of apelin mRNA are found in this tissue [14]. This lack of testicular APJ confirms previous findings in the rat by us [34] and others [17,30] using RT-PCR, but differs from other RT-PCR studies showing expression in human and mouse testis [30]. It is possible that testicular APJ is developmentally regulated since APJ mRNA expression appears to be higher in infant compared to adult peripheral tissues [17].

In this study we provide the first detailed characterization of APJ distribution in the mouse and report a clear correlation between mouse APJ transcription and translation. The APJ expressing tissues in the mouse where potential functional correlations are identified are the brain, heart, pituitary gland and adrenal gland. Expression was also observed in kidney, lung, stomach, uterus and ovary and no expression of APJ transcript or I^125^[Pyr^1^]apelin-13 binding could be observed in a number of tissues including liver and pancreas. We cannot discount the possibility that low levels of (possibly rapidly turning-over) APJ mRNA are below the detection threshold of ISHH, which may be detected with more sensitive methods such as RT-PCR, however it must be stressed that the functional significance of low levels of mRNA as detected by RT-PCR in the CNS or peripheral tissue samples is unknown. There appears to be a species difference in central APJ distribution and in the pituitary gland, with a widespread central APJ distribution in the rat compared to a more restricted distribution seen in the mouse, while APJ distribution in peripheral tissues appears to be comparable between rat and mouse. The functional significance of the apparent species differences in the central expression of APJ mRNA is not known. Our study suggests however that the apelin/APJ system may have a more wide-ranging central role in the rat than the mouse, that should be considered when drawing comparisons between studies in the rat and APJ KO mice.

## Figures and Tables

**Table 1:** Distribution of APJ mRNA and I^125^[Pyr^1^]apelin-13 binding/protein in mouse and rat tissues. Mouse data is relative distribution of APJ mRNA and I^125^[Pyr^1^]apelin-13 binding in the mouse. Analyses were made on four male and four female mice. Presented data is the subjective judgment of two observers. No apparent gender differences were detected. Rat data is consolidated from the following quantitative and semi-quantitative sources [9,10,16,17,22,29,30,53] using ISHH; Northern blot; RT-PCR; quantitative real time PCR; enzyme immuno-assay and immunohistochemistry.

Tissues	Mouse		Rat			
	APJ		APJ		Apelin	
	mRNA	I^125^[Pyr^1^]apelin-13 binding	mRNA	Protein	mRNA	Protein
Brain	−/+	−/+	+	+	++	+
-PVN	++	++	++	++	ND	ND
-SON	++	++	++	ND	ND	ND
Anterior pituitary	+++	+++	++/ + ++	ND	+/ + +^b^	+/ + +
Intermediate pituitary	−/+	−/+	−/+	ND	+/ + +^b^	−
Posterior pituitary	++	−/ + +	−/+	ND	+/ + +^b^	++
Thymus	−/+	+	−	ND	ND	ND
Lung	+++	++/+++	+++	++	++/+++	+++
Heart	+++	+++	++/+++	+/++	+/+++	+
Liver	−	−	−	ND	−/+	+
Adrenal gland	+++^a^	+++^a^	++	ND	−/+	+
Kidney	+/+++	+/+++	+/++	ND	+/++	+
Pancreas	−	−	+	ND	−	+
Spleen	−/+	−/+	−	ND	−	+
Stomach	+	+	+	+	+/+++	+/++
Intestine	++	++/+++	+	+	−/+	+
Bladder	+	+	−/+	ND	−	ND
Colon	+	+	+	+	−/+	+
Ovary	+++	+++	+/+++	+	++	+
Uterus	+++	+++	+/++	+	−/+	+
Mammary gland	−/+	++	+/++	+++	+++	+++
Testes	−	−	−	+	+/++	++

+++ = high; ++ = moderate; + = low level; − = absence of APJ mRNA or protein. ND = no published data on distribution.

a +++ expression only in adrenal cortex.

b Cumulative presence from analysis of whole pituitary.
